# Alterations of GABAergic Neuron-Associated Extracellular Matrix and Synaptic Responses in *Gad1*-Heterozygous Mice Subjected to Prenatal Stress

**DOI:** 10.3389/fncel.2018.00284

**Published:** 2018-09-05

**Authors:** Tianying Wang, Adya Saran Sinha, Tenpei Akita, Yuchio Yanagawa, Atsuo Fukuda

**Affiliations:** ^1^Department of Neurophysiology, Hamamatsu University School of Medicine, Hamamatsu, Japan; ^2^Department of Genetic and Behavioral Neuroscience, Graduate School of Medicine, Gunma University, Maebashi, Japan; ^3^Advanced Research Facilities and Services, Preeminent Medical Photonics Education and Research Center, Department of Genetic and Behavioral Neuroscience, Hamamatsu University School of Medicine, Hamamatsu, Japan

**Keywords:** prenatal stress, *Gad1* gene, perineuronal nets, dystroglycan, psychiatric disorders

## Abstract

Exposure to prenatal stress (PS) and mutations in *Gad1*, which encodes GABA synthesizing enzyme glutamate decarboxylase (GAD) 67, are the primary risk factors for psychiatric disorders associated with abnormalities in parvalbumin (PV)-positive GABAergic interneurons in the medial prefrontal cortex (mPFC). Decreased expression of extracellular matrix (ECM) glycoproteins has also been reported in patients with these disorders, raising the possibility that ECM abnormalities may play a role in their pathogenesis. To elucidate pathophysiological changes in ECM induced by the gene–environment interaction, we examined heterozygous GAD67-GFP (Knock-In KI; GAD67^+/GFP^) mice subjected to PS from embryonic day 15.0 to 17.5. Consistent with our previous study, we confirmed a decrease in the density of PV neurons in the mPFC of postnatal GAD67^+/GFP^ mice with PS, which was concurrent with a decrease in density of PV neurons surrounded by perineuronal nets (PNNs), a specialized ECM important for the maturation, synaptic stabilization and plasticity of PV neurons. Glycosylation of α-dystroglycan (α-DG) and its putative mediator fukutin *(Fktn)* in the ECM around inhibitory synapses has also been suggested to contribute to disease development. We found that both glycosylated α-DG and the mRNA level of *Fktn* were reduced in GAD67^+/GFP^ mice with PS. None of these changes were detected in GAD67^+/GFP^ naive mice or wild type (GAD67^+/+^) mice with PS, suggesting that both PS and reduced *Gad1* gene expression are prerequisites for these changes. When assessing the function of interneurons in the mPFC of GAD67^+/GFP^ mice with PS through evoked inhibitory post-synaptic currents (eIPSCs) in layer V pyramidal neurons, we found that the threshold stimulus intensity for eIPSC events was reduced and that the eIPSC amplitude was increased without changes in the paired-pulse ratio (PPR). Moreover, the decay rate of eIPSCs was also slowed. In line with eIPSC, spontaneous IPSC (sIPSC) amplitude, frequency and decay tau were altered. Thus, our study suggests that alterations in the ECM mediated by gene-environment interactions might be linked to the enhanced and prolonged GABA action that compensates for the decreased density of PV neurons. This might be one of the causes of the excitatory/inhibitory imbalance in the mPFC of psychiatric patients.

## Introduction

Prenatal stress (PS) is a risk factor that can change the trajectory of fetal brain development and have long-term effects on adult brain function, which may result in psychiatric disorders like autism spectrum disorder (ASD), depression and schizophrenia (Bock et al., 2015). Animal model studies have demonstrated that PS significantly alters neural circuit development and may produce behavioral deficits suggestive of psychiatric disorders (Weinstock, 2008) which is regulated by excitatory/inhibitory balance (E/I balance) at synaptic and network levels. One of the key molecules that regulates E/I balance in the brain is the inhibitory neurotransmitter γ-aminobutyric acid (GABA). Early in development, GAD67, an isoform of GABA-synthesizing enzymes, is already expressed, and synthesized GABA has an excitatory effect that regulates neuronal migration and maturation (Ben-Ari, 2002; Heng et al., 2007; Wang and Kriegstein, 2009; Wang et al., 2014; Watanabe and Fukuda, 2015). During the postnatal period, GABAergic responses undergo a switch from being excitatory to inhibitory. Dysfunction of GABAergic signaling results in E/I imbalance affecting individual synaptic inputs to a neuron and overall neural circuitry suggest that reduced GABA-mediated signaling might also be a risk factor for psychiatric disorders. To determine whether PS and GABA reduction may interact and worsen neural development, we previously examined (GAD67-green fluorescent protein (GFP) Knock-In (KI) GAD67^+/GFP^) mice, in which one *Gad1* gene is replaced with the GFP gene to reduce GABA production by half (Tamamaki et al., 2003; Wang et al., 2009). Using these mice, we reported that the application of restraint PS through mother mice suppressed the neurogenesis of GABAergic neurons in the medial ganglionic eminence (MGE) of GAD67^+/GFP^ embryos. Furthermore, this resulted in the reduced density of parvalbumin (PV)-expressing GABAergic neurons in the medial prefrontal cortex (mPFC) of postnatal GAD67^+/GFP^ mice, but not of wild type littermates (Uchida et al., 2014). The vulnerability of GAD67^+/GFP^ mice to PS was also reflected in their altered corticosterone levels (Uchida et al., 2011). Thus, our studies identified a gene (*Gad1*)–environment (PS) interaction related to the development of PV-positive interneurons. PV interneurons in the neocortex are fast-spiking (FS) and exert strong inhibitory control on cortical pyramidal neurons, which may be reflected in gamma oscillations of cortical networks (Somogyi and Klausberger, 2005). Dysfunction of PV interneurons has been suggested to cause impaired neuronal synchrony, leading to altered sensory perception, and social and cognitive deficits, i.e., the symptomatology of psychiatric disorders (Hashimoto et al., 2003; Spencer et al., 2004; Gogolla et al., 2009b; Carlson et al., 2011; Wang et al., 2011; Lewis et al., 2012; Fung et al., 2014; Lovett-Barron and Losonczy, 2014). Thus, the E/I imbalance caused by the maldevelopment and dysfunction of PV interneurons has been widely implicated in the pathophysiology of various psychiatric disorders (Gogolla et al., 2009b; Lewis et al., 2012; Chung et al., 2016). However, the mechanism of interaction between PS and GABA reduction during development that causes PV interneuron dysfunction has not been elucidated.

Perineuronal nets (PNNs), a well-organized proteoglycan component of the extracellular matrix (ECM), enwrap the cell soma and proximal neurites of PV neurons in a lattice-like fashion (Härtig et al., 1992). PNNs function to maintain the optimal local homeostasis of ions, protect against oxidative stress, and mediate the opening and closing of the critical period of cortical plasticity (Brückner et al., 1993; Härtig et al., 1999; Pizzorusso et al., 2002; Wang and Fawcett, 2012; Cabungcal et al., 2013). Several studies have indicated that PNNs are essential for learning and memory (Gogolla et al., 2009a; Romberg et al., 2013). Decrements of PNNs in multiple brain regions were found in subjects with schizophrenia, suggesting that PNNs might play a role in its pathogenesis (Pantazopoulos et al., 2010, 2015; Mauney et al., 2013; Enwright et al., 2016). Another component of ECM associated with inhibitory synapses, dystroglycan, is a central member of the dystrophin glycoprotein complex (DGC) and has been implicated in the maintenance of mature inhibitory synapses (Lévi et al., 2002). Its dysfunction leads to various muscular dystrophies categorized as dystroglycanopathies, which are caused by mutations in genes involved in the O-linked glycosylation of α-dystroglycan (α-DG). A regular glycosylation pattern of α-DG is essential for the binding of its extracellular ligands such as laminin, agrin, perlecan and neurexin (Ervasti and Campbell, 1993; Gee et al., 1994; Talts et al., 1999; Sugita et al., 2001). Mutations in enzymes glycosylating α-DG result in muscular dystrophies associated with cognitive and neurological deficits in mice models, partially replicating the symptoms observed in patients, thus corroborating the causative role of aberrant α-DG glycosylation in the abnormal development of the central nervous system (Messina et al., 2010; Waite et al., 2012; Comim et al., 2014). Taken together, the GABAergic neuron-associated ECM affects diverse processes of nervous system development and neural function.

Here, we tested the hypothesis that our gene–environment interaction model causes abnormalities in the ECM and functions of GABAergic interneurons in the mPFC of postnatal mice. We found that the proportion of PV neurons associated with PNNs was decreased in the mPFC of GAD67^+/GFP^ mice subjected to PS. Both the cluster area of glycosylated α-DG and the mRNA level of its putative glycosylating enzyme fukutin *(Fktn)* were reduced. Electrophysiological analysis indicated lower stimulus threshold intensity and increased amplitude of evoked inhibitory post-synaptic currents (eIPSCs) with prolonged decay rates in layer V pyramidal neurons in the mPFC. In addition, spontaneous IPSC (sIPSC) amplitude, frequency and decay tau were altered. These findings suggest a possible link between the altered ECM and enhanced GABA actions that compensate for the decreased density of PV neurons.

## Materials and Methods

### GAD67-GFP Knock-in Mice

The GAD67-GFP (Δneo) transgenic mouse, referred to hereafter as the GAD67-GFP Knock-In (KI) mouse, expresses enhanced GFP under the regulation of the endogenous *Gad1* promoter (Tamamaki et al., 2003). The heterozygous GAD67-GFP KI mice showed 50% of GAD67 replaced by GFP. Although the possibility of cellular toxicity of GFP has been reported (Ansari et al., 2016), all of parameters evaluated in our previous study did not show significant difference between heterozygote and wild type (Uchida et al., 2014), indicating toxicity of GFP to cells would not affect our final results. These mice were bred on a C57BL/6N background. In the present study, female GAD67^+/+^ mice (Japan SLC, Hamamatsu, Japan) were placed with male (>9 weeks) GAD67^+/GFP^ mice overnight in a cage under a 12-h light–dark cycle lights off from 19:00 to 07:00). The day when a vaginal plug was identified was defined as embryonic day (E) 0. All procedures were in accordance with guidelines issued by the Hamamatsu University School of Medicine on the ethical use of animals for experimentation and were approved by the Committee for Animal Care and Use (No. 2016029). All efforts were made to minimize the number of animals used and their suffering.

### Maternal Restraint-and-Light Stress

The stress procedure was performed three times a day for 45 min per session (08:30–09:15, 12:30–13:15 and 16:30–17:15) from E15.0 to E17.5 using a transparent plastic tube with a diameter of 3 cm. The plastic tube was placed 50 cm under two halogen lights (150 W each). Newborns in both control and stress group were raised by naive surrogate mothers with the same delivery date at postnatal day (P) 0 and bred until P21 with their surrogate mother. We referred to GAD67^+/GFP^ mice with and without stress as HT-PS and HT-CTRL, respectively.

### Immunohistochemistry

**Figure 1:**
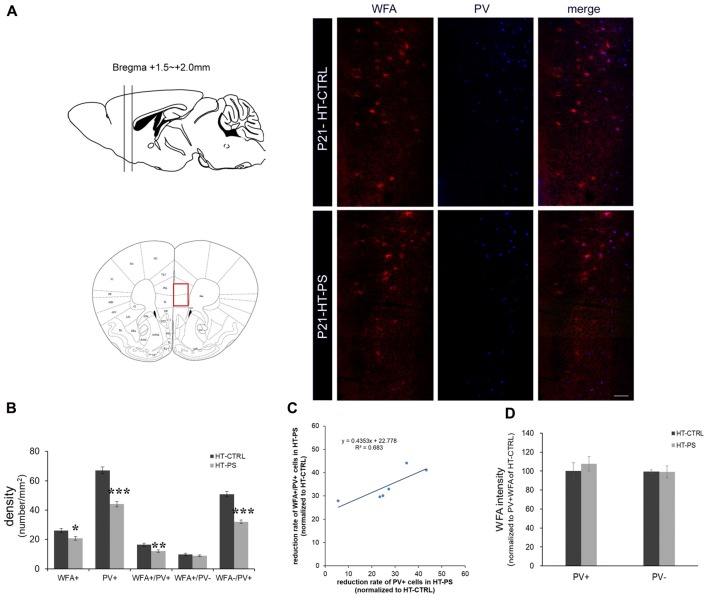
Density of perineuronal nets (PNNs) is decreased in the medial prefrontal cortex (mPFC) of glutamate decarboxylase (GAD)67^+/GFP^ offspring with prenatal stress (PS). **(A)** Coronal slices were collected in the prefrontal area (Bregma +1.5 mm to +2.0 mm) and images were obtained from the medial prefrontal area indicated by a red rectangle (left). Representative photomicrographs show the distribution of Wisteria Floribunda Agglutinin (WFA)-labeled PNNs in the mPFC. Fluorescence images of a coronal section in control GAD67^+/GFP^ offspring and those with PS at P21 immunostained with WFA (red) and (parvalbumin PV, blue). **(B)** Quantitative analysis of cell density in the mPFC.WFA+ and WFA+/PV+ cells were significantly decreased in mPFC in subjects with PS compared with controls (HT-CTRL: 22 slices; HT-PS: 17 slices from six mice, *t*-test, *P < 0.05; **P < 0.01). The number of WFA−/PV+ cells was also significantly decreased in mPFC in subjects with PS (*t*-test, ***P < 0.001). No significant differences were detected in the number of WFA+/PV− in the mPFC between the two groups (*t*-test, *P* = 0.344). **(C)** The reduction rate of PV+ and WFA+/PV+ cells in the mPFC of HT-PS were normalized to the average value obtained from HT-CTRL. Regression analyses revealed a significant linear relationship between reduction rate of PV+ cells and reduction rate of WFA+/PV+ cells in the mPFC of HT-PS (*R*^2^ = 0.683, *P* < 0.01). **(D)** Quantitative analysis of fluorescence intensity of WFA in the mPFC of control and stressed mice. There are no significant differences in WFA intensity between subjects with PS (102 PV+ cells and 78 PV− cells from six mice) and normal control subjects (93 PV+ cells and 82 PV− cells from six mice, 2-tailed *t*-test). Scale bars: 100 μm. Error bars represent the SEM.

P21 male mice were perfused transcardially with cold saline, followed by a freshly prepared solution of 4% paraformaldehyde (PFA) in 0.1 M phosphate buffer (PB), pH 7.4. Mice brains were rapidly removed and post fixed overnight in 4% PFA/0.1 M PB at 4°C. Twenty-five-micrometer thick coronal sections were cut with a cryostat (HM560R; Zeiss Microm, Walldorf, Germany) and collected in cold 0.1 M PB. For immunohistochemistry, sections were blocked for 1 h in 10% (*v/v*) normal goat serum in 0.1 M PB with 0.2% Triton X-100 at room temperature (RT) and then incubated with primary antibodies overnight at 4°C. The following primary antibodies were used: biotinylated lectin Wisteria Floribunda Agglutinin (WFA, 1:500; Vector Laboratories, Burlingame, CA, USA), aggrecan (1:1,000; Millipore, Billerica, MA, USA), PV (ACAN; 1:1,000; Sigma Chemical Co., St. Louis, MO, USA), α-dystroglycan clones IIH6C4 (1:1,000; Millipore, Temecula, CA, USA), and GFP rabbit (1:1,000; Molecular Probes, Eugene, OR, USA). The sections were then washed with 0.1 M PB and incubated with secondary antibodies diluted in blocking buffer (Alexa Fluor 488 anti-rabbit IgG, Alexa Fluor 594 anti-mouse IgG, Alexa Fluor 594 anti-mouse IgM or Alexa Fluor 405 streptavidin-conjugated antibody; 1:1,000; Molecular Probes) for 2  h at RT. The sections were washed with 0.1 M PB, mounted on gelatin-coated slides, and observed using a confocal microscope (Olympus FV-1,000, Tokyo, Japan) or an inverted microscope system (BZ-9,000, KEYENCE, Osaka, Japan). The images were analyzed using Imaris software package (Bitplane, Switzerland). The fluorescence signals were defined based on a threshold (20%–40% of maximal intensity). The WFA (or ACAN) surrounding PV neuron was detected using the Contour Surface function of Imaris software that creates a new channel and applies a mask within the channel. Masks were also configured for WFA positive but PV-negative cells. The intensity of the WFA signal contained in the masks for PV-positive or PV-negative was determined by subtracting the background intensity from the total fluorescence intensity. The intensity values were normalized to the mean intensity value of the WFA (or ACAN) surrounding the PV neurons of HT-CTRL mice. The IIH6C4 clusters were also analyzed with the Contour Surface function of Imaris. The minimum diameter of cluster was determined by using the point-to-point measurement function to measure the diameter of the smallest cluster found in the image under the slice view. The areas of individual IIH6C4+ clusters were determined and the cluster density was expressed by the ratio of surface area of IIH6C4 cluster over the background area of 1 mm^2^. The quantitative analysis of immunostaining was performed as described previously (Uchida et al., 2014). In brief, to ensure unbiased counting, the regions of interest (ROI) of mPFC (framed area in Figure 1A) containing prelimbic and infralimbic cortex were consistently captured using 20× lens. This framing was performed to avoid double counting of positive signal. Two to four coronal sections from each mouse brain (spaced by 100–200 μm, located at about +1.5 mm to +2.0 mm from Bregma) containing the mPFC were used. These conditions were used for all quantitative analysis. The different cell types were recognized by a software algorithm based on predefined criteria that disregards noise outside of the fluorescence intensity window. These conditions were saved and consistently applied for all subsequent cell counting assays for all groups. These randomizations for quantitative assays were rigorously adhered to during analysis.

### RNA Extraction and Quantitative RT-PCR

The mPFC was dissected from P21 male mice after anesthesia with isoflurane. Total RNA samples from each genotype were prepared using an RNeasy RNA extraction kit (Qiagen, Chatsworth, CA, USA). RNA concentrations were determined using a Nanodrop ND-1,000 (Thermo Fisher Scientific Inc., Wilmington, DE, USA) UV-Vis spectrophotometer. Primers were designed using Primer-BLAST^1^ and verified by BLAST^2^. Highly purified salt-free primers for the target gene *Fktn* (forward primer, 5′-GCAACTACCTCTGGCATGGT-3′; reverse primer, 5′-ATGTACTGCTGGAGGAACGC-3′) and for reference gene *Gapdh* (forward primer, 5′-TGTGTCCGTCGTGGATCTGA-3′; reverse primer, 5′-TTGCTGTTGAAGTCGCAGGAG-3′) were generated commercially (Rikaken Co. Ltd., Tokyo, Japan). First-strand complementary DNA (cDNA) was generated from total RNA samples using the random hexamer primers and SuperScript IV reverse transcriptase (Invitrogen, Breda, Netherlands) according to the manufacturer’s recommended protocol. Real Time PCR reactions were carried out using a Thermal Cycler Dice^®^ Real Time System (Takara Bio Inc., Otsu, Japan). *Fktn* transcript expression was normalized to the housekeeping gene *Gapdh* in triplicate for each sample (ΔCt). Individual ΔCt values were normalized to a littermate control sample (ΔΔCt) and converted from log phase to determine the ratio of gene expression relative to WT-CTRL. We consistently monitored the time and methods for tissue sample processing, storage conditions, amount of total RNA (1,000 ng) and the mean difference between three technical replicates. The raw threshold cycle (C_T_) values for *Gapdh* ranged from 12.76 to 13.63, with the mean C_T_ values for all tested samples being 13.18 for HT-CTRL, 13.10 for HT-PS, 13.07 for WT-CTRL and 13.09 for WT-PS. Therefore, *Gapdh* as a reference gene for qRT-PCR using rodent brain with restraint stress was verified as reported previously (Uchida et al., 2010; Seo et al., 2017).

### Slice Preparation and Electrophysiology

P19–P23 old male mice were anesthetized with isoflurane and decapitated. The brains were quickly removed and placed into ice-cold oxygenated, modified artificial cerebrospinal fluid (ACSF; in mM, 220 sucrose, 2.5 KCl, 1.25 NaH_2_PO_4_, 12.0 MgSO_4_, 0.5 CaCl_2_, 26.0 NaHCO_3_ and 30.0 glucose at pH 7.4). Coronal mPFC slices with a thickness of 350 μm were prepared in modified ACSF using a vibratome (Campden Instruments, Loughborough, Leicestershire, UK). Slices were allowed to recover for 90 min on nylon meshes (with 1 mm pores) placed on dishes and submerged in standard ACSF consisting of (in mM): 126 NaCl, 2.5 KCl, 1.25 NaH_2_PO_4_, 2.0 MgSO_4_, 2.0 CaCl_2_, 26.0 NaHCO_3_ and 20.0 glucose, saturated with 95% O_2_/5% CO_2_ at RT. Slices were then transferred to an imaging chamber on the stage of an upright microscope (BX51WI; Olympus Tokyo, Japan) and continuously perfused with oxygenated ACSF at a flow rate of 2 mL/min and at a temperature of 30°C. Whole-cell patch-clamp recordings were made from layer V pyramidal cells of mPFC and voltage clamped at −70 mV. The electrode resistance ranged from 3 Ω to 5 MΩ when the electrode was filled with a solution containing (in mM) 150 CsCl, 2.0 MgCl_2_, 10 HEPES, 1.0 EGTA, 3.0 Na_2_ATP, 0.2 Na_2_GTP and 5.0 QX-314 bromide (Tocris, Ellisville, MO, USA) with pH maintained at 7.3 and an osmolarity of 303 mOsm. The reversal potential of chloride ion (E_Cl_) was calculated to be −3.9 mV. To elicit eIPSC, a platinum-iridium bipolar electrode was placed <100 μm from the recorded cell and events were evoked by a 0.2 Hz pulse train with stimuli of 10–100 μA. The recordings were performed in the presence of CNQX (10 μM), D-AP5 (50 μM) and GABA_B_ receptor blocker CGP55845 (3 μM). Liquid junction potential was measured to be 4.6 mV and was corrected for recordings online. Membrane currents were recorded by a multiclamp 700B amplifier (Axon Instruments, Sunnyvale, CA, USA) with a Bessel prefilter at 2 KHz and digitized at 10 KHz using a Digidata 1440A data-acquisition system (Axon instruments, Sunnyvale, CA, USA). Series resistance (Rs) was compensated by 70%. Cells with Rs <25 MΩ were used for analysis. To evaluate stimulus-response functions, eIPSC amplitude was measured with five events averaged for each neuron per stimulation intensity. Paired-pulse ratio (PPR; eIPSC2/eIPSC1) was estimated at 50, 80 and 250 ms interstimulus intervals (ISI). The maximal eIPSC response for each neuron was used to calculate the 60%–70% of maximal stimulus intensity for rise time and decay rate analysis. Ten eIPSC events were elicited using this stimulus intensity. Rise time was estimated as the elapsed time between 10% and 90% of the peak amplitude. To estimate decay tau values, the decay phase of each event was visually inspected for any contamination by sIPSC and miniature IPSC (mIPSC) events. Only evoked responses with a uniform decay phase were subjected to double exponential fitting from the peak of the eIPSC to a cutoff window depending on the recovery to baseline current level. Following fitting, each fit was tested for the goodness of fit using correlation values (R-square method). Based on this analysis algorithm, two decay tau values (τ_fast_ and τ_slow_) were estimated. In addition, percentage of relative amplitude components (Rel. amp._fast_ and Rel. amp._slow_) of each eIPSC fitted by corresponding τ_fast_ and τ_slow_ were calculated. sIPSC were recorded from the HT-CTRL and HT-PS groups in the presence of ionotropic glutamate receptor antagonists CNQX (10 μM), APV (50 μM) and GABA_B_ receptor antagonist CGP 55845 (3 μM). For analysis of sIPSC, three non-overlapping epochs of 60 s each were selected and threshold-based event detection was performed with threshold set at three times standard deviation (3× SD) of baseline noise. The events from equal no. of cells were then plotted using cumulative probability function for sIPSC amplitude, decay tau and interevent interval.

### Statistical Analysis

The density of immunopositive cells in each area, and the fluorescence intensities of WFA+ PNNs and aggrecan-positive (ACAN+) PNNs in P21 mice brain were analyzed using the *t*-test or one-way analysis of variance (ANOVA) with appropriate* post hoc* tests. The density of clusters positive for the glycosylated pattern of α-DG was analyzed using one-way ANOVA followed by *post hoc* Ryan-Einot-Gabriel-Welsch *F* test. The qRT-PCR of *Fktn* was analyzed by the Kruskal-Wallis test followed by stepwise stepdown multiple comparisons. The mean of decay tau, rise time and PPR of eIPSCs parameters were analyzed by *t*-test. The stimulus-amplitude relationship was analyzed by the Mann-Whitney *U*-test. The percentages of rel. amp._fast_ and rel. amp_slow_ were compared using the *t*-test. For sIPSC events analysis, distributions for amplitude, decay tau and interevent interval were compared using Kolmogorov-Smirnov (K-S) test. A value of *P* < 0.05 was considered statistically significant.

## Results

### Density of Perineuronal Nets Is Decreased in the mPFC of GAD67^+/GFP^ Offspring With Prenatal Stress (PS)

To evaluate PNN alterations in the (mPFC) of our gene–environment interaction model, we applied (Wisteria Floribunda Agglutinin (WFA), a frequently used lectin that specifically detects N-acetylgalactosamine of polysaccharide glycosaminoglycan (GAG) chains of chondroitin sulfate proteoglycans (CSPGs) to label the PNNs. PV and WFA double staining was performed (Figure 1). Approximately 60% of WFA-labeled PNNs surrounded PV+ cells, while 40% of WFA-labeled PNNs surrounded PV− cells. The cell density analysis was carried out using six animals from each group. GAD67^+/GFP^ mice subjected to PS showed a significant loss of WFA-labeled PNNs (in cells/mm^2^; HT-CTRL: 26.10 ± 1.48; HT-PS: 20.74 ± 1.26; *P* < 0.05 by *t*-test; Figures 1A,B). Consistent with our previous study, numbers of PV+ GABAergic interneurons were significantly less in the mPFC of HT-PS (in cells/mm^2^; HT-CTRL: 67.10 ± 2.22; HT-PS: 44.07 ± 1.83; *P* < 0.001 by *t*-test). The density of WFA enwrapping PV+ neurons in the mPFC of GAD67^+/GFP^ mice with PS was significantly lower in comparison with controls (in cells/mm^2^; HT-CTRL: 16.32 ± 1.06; HT-PS: 11.99 ± 0.85; *P* < 0.01 by *t*-test). The density of PV+ neurons without PNN enwrapping was also found to be significantly reduced compared with controls (in cells/mm^2^; HT-CTRL: 50.77 ± 1.82; HT-PS: 32.07 ± 1.22; *P* < 0.001 by *t*-test; Figure 1B). However, the fraction of PV− cells with WFA showed a similar distribution in stress (in cells/mm^2^; 9.77 ± 0.84 in HT-CTRL; 8.75 ± 0.58 in HT-PS;* P* = 0.344 by *t*-test). Further evaluation of this fraction to estimate the number of PV−/GFP+ GABA cells and GFP− non-GABA cells associated with WFA, revealed no significant differences (Supplementary Figure S1). Regression analysis indicated a significant positive correlation between the reduction rate of PV+ cells and reduction rate of WFA+/PV+ cells in the mPFC of HT-PS (Figure 1C; *R*^2^ = 0.683; *P* < 0.01). These results suggest the reduction of PNN is PV neuron selective. In addition, we examined the fluorescence level of WFA and found the fluorescence intensity of WFA immunoreactivity surrounding PV+ and PV− cells were equivalent (Figure 1D).

**Figure 2:**
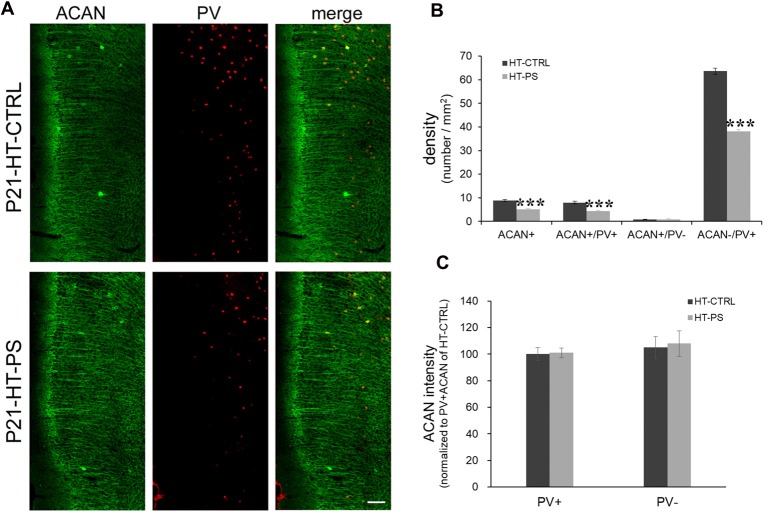
Density of aggrecan-positive (ACAN+) cells is decreased in the mPFC of GAD67^+/GFP^ offspring with PS. **(A)** Representative photomicrographs showing the distribution of ACAN-labeled perineuronal nets in the mPFC. Fluorescent images of a coronal section in control GAD67^+/GFP^ offspring (up) and those with (PS; down) at P21 immunostained with ACAN (green) and (PV; red). **(B)** Quantitative analysis of ACAN+, ACAN+/PV+, ACAN−/PV+ and ACAN+/PV− cells in the mPFC. The number of ACAN+, ACAN+/PV+ and ACAN−/PV+ cells were significantly decreased in the mPFC of subjects with PS compared with controls (HT-CTRL: 17 slices; HT-PS: 13 slices from six mice; *t*-test, ***P < 0.001). There are no significant differences in the number of ACAN+/PV− cells between subjects with PS and normal control subjects. **(C)** Quantitative analysis of fluorescence intensity of ACAN in the mPFC of control and stressed mice. There are no significant differences in ACAN intensity between subjects with PS (66 PV+ cells and 13 PV− cells from six mice) and normal control subjects (68 PV+ cells and 16 PV− cells from six mice, 2-tailed *t*-test). Scale bars: 100 μm. Error bars represent the SEM.

The lectican family of CSPGs, such as ACAN, brevican, neurocan, phosphacan and versican, are the main components of PNNs (Yamaguchi, 2000). We examined the distribution of ACAN-based PNNs around PV-expressing GABAergic neurons in the medial regions of the mouse PFC (Figure 2). Consistent with the result of WFA-labeling PNNs, aggrecan-positive (ACAN+) PNNs were significantly less in the mPFC of mice with double-hits compared with that in control subjects (in cells/mm^2^; HT-CTRL: 8.81 ± 0.50; HT-PS: 5.05 ± 0.42; *P* < 0.001 by *t*-test; Figure 2B). Among these cells, ACAN+ PNNs surrounding PV+ cells (in cells/mm^2^; ACAN+/PV+ cells; HT-CTRL: 7.99 ± 0.55; HT-PS: 4.29 ± 0.44; *P* < 0.001 by *t*-test) were decreased. The density of ACAN+/PV− cells (in cells/mm^2^; HT-CTRL: 0.82 ± 0.19; HT-PS: 0.76 ± 0.24; *P* = 0.866 by *t*-test) did not change in the mPFC of GAD67^+/GFP^ offspring with PS compared with controls. In our analysis some slices have no ACAN+/PV− cells in the ROI (HT-CTRL: 7 out of 16 slices; HT-PS: 7 out of 13 slices; WT-CTRL: 8 out of 16 slices; WT-PS: 6 out of 13 slices). Loss of ACAN−/PV+ was also detected in GAD67^+/GFP^ offspring with PS (in cells/mm^2^; HT-CTRL: 63.62 ± 1.26; HT-PS: 38.20 ± 0.75; *P* < 0.001 by *t*-test). The very low numbers of ACAN+/PV− cells as compared to the ACAN+/PV+ cells in the same ROI encompassing equal areas in all the groups suggests that PNNs composed of ACAN were mostly associated with PV neurons in the mPFC. Therefore, a selective reduction in the ACAN+/PV+ cells in HT-PS further corroborates the PNNs reduction quantified by means of WFA staining in Figure 1. No significant differences in the fluorescence intensity of ACAN immunoreactivity surrounding PV+ and PV− cells were detected between the two groups (Figure 2C).

### Unchanged Densities of PNNs in the mPFC of Mice With Either PS or *Gad1* Heterozygosity Alone at P21

**Figure 3:**
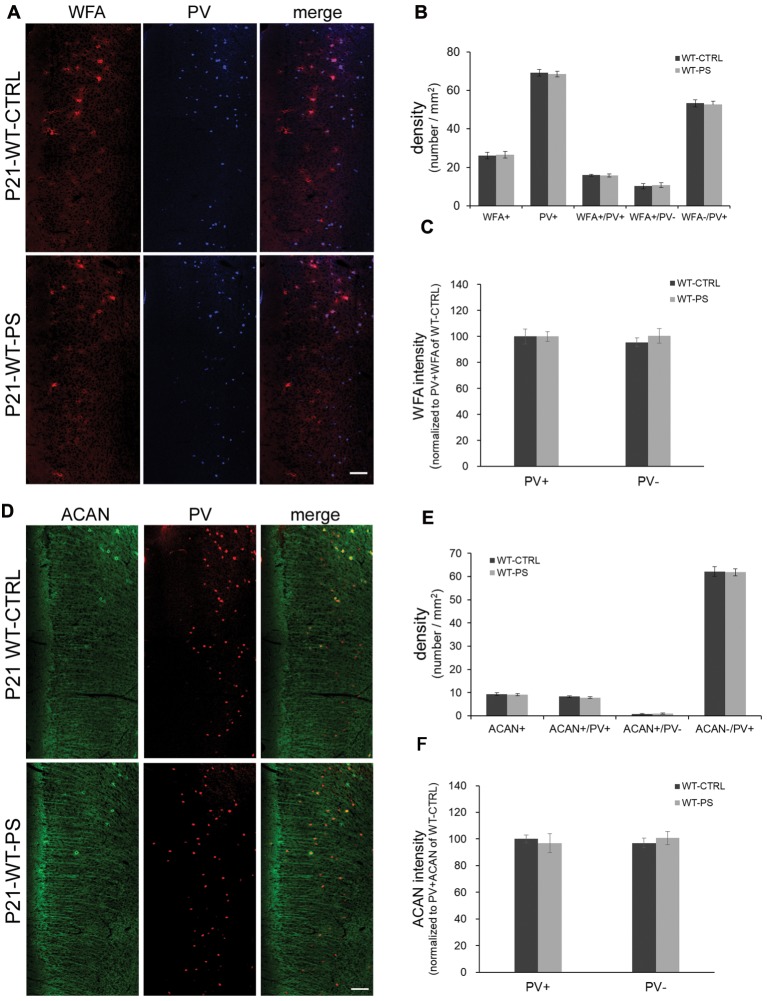
Unchanged densities of PNNs in the mPFC of prenatally stressed GAD67^+/+^ mice at P21. **(A)** Representative immunostaining images of WFA (red) and (PV; blue) from coronal sections of P21 wild type (GAD67^+/+^) control and stressed brains. **(B,C)** Quantitative analysis of cell densities **(B)** and fluorescence intensity of WFA **(C)** in the mPFC. Neither the cell densities (16 slices from six mice in each group; 2-tailed *t*-test) nor the WFA intensities (WT-CTRL, 84 PV+ cells and 93 PV− cells from six mice; WT-PS, 74 PV+ cells and 76 PV− cells from six mice; 2-tailed *t*-test) showed significant differences between normal control and PS mice. **(D)** Representative immunostaining images of ACAN (green) and PV (red) from coronal sections of GAD67^+/+^ control and stressed brains. **(E,F)** Quantitative analysis of cell densities **(E)** and fluorescence intensity of ACAN **(F)** in the mPFC. Neither cell densities (WT-CTRL, 16 slices from six mice; WT-PS, 14 slices from six mice; 2-tailed *t*-test) nor the WFA intensities (WT-CTRL, 63 PV+ cells and 18 PV− cells from six mice; WT-PS, 62 PV+ cells and 16 PV− cells from six mice; 2-tailed *t*-test) showed significant differences between the normal control and PS groups. Scale bars: 100 μm. Error bars represent the SEM.

To address the question of whether PS or *Gad1* gene mutation alone contributes to the selective reduction of PNNs-associated PV+ neurons, we examined the density of PNN-labeling in the mPFC of GAD67^+/+^ offspring with PS using WFA and ACAN staining (Figure 3). In striking contrast to GAD67^+/GFP^ mice, wild type mice subjected to PS did not show the loss of WFA-labeled PNNs (Figures 3A,B). The density of PV+ interneurons in WT-CTRL and WT-PS were similar with that in HT-CTRL mice. No differences in the densities of individual WFA+/PV+, WFA+/PV− and WFA−/PV+ cells in the mPFC were found between the control and stressed groups (Figure 3B). The fluorescence intensity of WFA immunoreactivity surrounding PV+ and PV− cells was also similar in WT-CTRL and WT-PS (Figure 3C). Similar with that of WFA+ PNNs, GAD67^+/+^ mice did not show a loss of ACAN-labeled PNNs following PS (Figures 3D,E). Neither the cell density nor the immunofluorescence intensity of ACAN was altered in WT-CTRL and WT-PS (Figures 3E,F). To further ascertain the effects of genotype on the fraction of cell subtypes, comparisons of multiple groups by using Dunnett’s test was performed. The cell densities of all cell subtypes in mPFC of HT-CTRL mice were similar to those in the WT-CTRL and WT-PS mice (Table 1). These data suggest that PS or mutation of the *Gad1* gene alone is not sufficient to induce the loss of PV cells and their surrounding PNNs.

**Table 1:** Descriptive statistics of cell density in medial prefrontal cortex (mPFC) and the corresponding statistical analysis for comparisons of means among four different groups.

Cell type	Group	Cell density mean ± SEM	ANOVA	*Post hoc* Dunnett’s test *p* value (vs. HT-CTRL)
PV+	HT-CTRL	67.10 ± 2.22	
	HT-PS	44.07 ± 1.83	0.000	0.000
	WT-CTRL	69.20 ± 1.69		0.759
	WT-PS	68.43 ± 1.51		0.918
WFA+	HT-CTRL	26.10 ± 1.48	
	HT-PS	20.74 ± 1.26	0.046	0.058
	WT-CTRL	26.11 ± 1.63		1.000
	WT-PS	26.45 ± 1.73		0.997
WFA+/PV+	HT-CTRL	16.32 ± 1.06	
	HT-PS	11.99 ± 0.85	0.004	0.003
	WT-CTRL	15.90 ± 0.39		0.967
	WT-PS	15.76 ± 0.80		0.928
WFA+/PV−	HT-CTRL	9.77 ± 0.84	
	HT-PS	8.75 ± 0.58	0.618	0.837
	WT-CTRL	10.20 ± 1.41		0.983
	WT-PS	10.69 ± 1.21		0.872
WFA−/PV+	HT-CTRL	50.77 ± 1.82	
	HT-PS	32.07 ± 1.22	0.000	0.000
	WT-CTRL	53.29 ± 1.85		0.602
	WT-PS	52.67 ± 1.83		0.773
ACAN+	HT-CTRL	8.81 ± 0.50	
	HT-PS	5.05 ± 0.42	0.000	0.000
	WT-CTRL	9.34 ± 0.57		0.793
	WT-PS	9.11 ± 0.48		0.949
ACAN+/PV+	HT-CTRL	7.99 ± 0.55
	HT-PS	4.29 ± 0.44	0.000	0.000
	WT-CTRL	8.28 ± 0.41		0.938
	WT-PS	7.86 ± 0.34		0.994
ACAN+/PV−	HT-CTRL	0.82 ± 0.19
	HT-PS	0.76 ± 0.24	0.932	0.997
	WT-CTRL	0.82 ± 0.26		1.000
	WT-PS	0.97 ± 0.24		0.938
ACAN−/PV+	HT-CTRL	63.62 ± 1.26
	HT-PS	38.20 ± 0.75	0.000	0.000
	WT-CTRL	62.16 ± 2.03		0.823
	WT-PS	61.82 ± 1.49		0.718

*Data are expressed as the mean ± SEM. Statistical analysis was performed by one-way ANOVA with *post hoc* Dunnett’s test (vs. HT-CTRL)*.

### Decrease in Glycosylated Pattern of α-Dystroglycan in the mPFC of GAD67^+/GFP^ Offspring With PS

**Figure 4:**
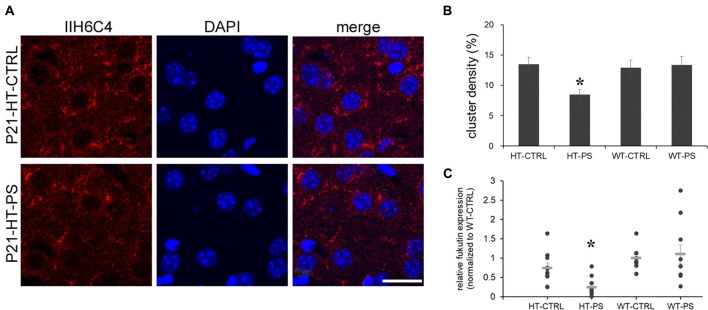
Decrease in glycosylated pattern of α-dystroglycan (α-DG) mediated by fukutin (*Fktn*) is detected in the mPFC of GAD67^+/GFP^ offspring with PS. **(A)** Coronal sections of mPFC in control (HT-CTRL, up) prenatally stressed (HT-PS, bottom) GAD67^+/GFP^ offspring at P21 immunostained with glycosylated α-dystroglycan (IIH6C4, red) and DAPI (blue). Glycosylation of α-DG was decreased in the mPFC of the PS group. **(B)** Quantification of the area of clusters positive for the glycosylated pattern of α-DG. Data are reported as the means ± SEM acquired from multiple slices from at least three mice per group (HT-CTRL, 16 slices; HT-PS, 15 slices; WT-CTRL, 16 slices; WT-PS, 18 slices from six mice in each group). The density of glycosylated α-DG in the mPFC of HT-PS mice was significantly lower than in the other three groups (*P < 0.05, one-way analysis of variance (ANOVA) followed by *post hoc* Ryan-Einot-Gabriel-Welsch *F*-test). **(C)** Individual data of *Fktn* mRNA expression levels were normalized to GAPDH and to the wild type control in each blot. The mean value (gray horizontal line) of HT-PS was significantly decreased compared with WT-CTRL and WT-PS mice (*P < 0.05, Kruskal-Wallis test;* n* = 10 mice in each group). Scale bars: 20 μm.

The central component of the DGC, α-DG, is implicated in brain development, synapse formation and plasticity (Waite et al., 2012), and particularly in the maintenance of inhibitory synapses (Lévi et al., 2002). Moreover, changes in the glycosylation of α-DG result in muscular dystrophies are often associated with cognitive and neurological deficits (Godfrey et al., 2011). To address whether gene–environment interactions may affect the functionality of α-DG, we carried out immunofluorescence staining with antibody IIH6C4. This antibody recognizes an O-mannosyl glycoepitope on α-DG. Thus, IIH6C4 binding would indicate a regular pattern of glycosylation of α-DG. IIH6C4 immunofluorescence showing perisomatic clusters was readily observed in mPFC at P21. Similar clustered distributions were obtained in the mPFC of WT-CTRL and WT-PS (data not shown). In the mPFC of HT-PS mice, IIH6C4 immunofluorescence was significantly reduced (Figure 4A). Quantitative analysis showed the IIH6C4+ cluster density in the mPFC of HT-PS mice was 8.5 ± 0.8%, which was significantly decreased compared with the other three groups (in %; HT-CTRL: 13.5 ± 1.1; WT-CTRL: 12.9 ± 1.2; WT-PS: 13.3 ± 1.4; *P* < 0.05, one-way ANOVA followed by *post hoc* Ryan-Einot-Gabriel-Welsch *F*-test; six mice in each group; Figure 4B).

As the IIH6C4 immunostaining showed the decrease in glycosylation of α-DG, we investigated one of the putative glycosyltransferases, *Fktn*, whose mutation results in the defective glycosylation of α-DG. We examined the expression of *Fktn* mRNA by quantitative PCR. In the mPFC of HT-PS, *Fktn* mRNA was significantly decreased compared with the other three groups (*P* < 0.05, Kruskal-Wallis test followed by stepwise stepdown multiple comparisons; Figure 4C). Therefore, the disruption of *Fktn* mRNA expression may culminate in a reduction of the glycosylated pattern of α-DG in GAD67^+/GFP^ mice with PS.

### Altered Excitability of the Inhibitory Network and Slower Decay Kinetics of Evoked IPSC in GAD67^+/GFP^ Mice With Prenatal Stress

**Figure 5:**
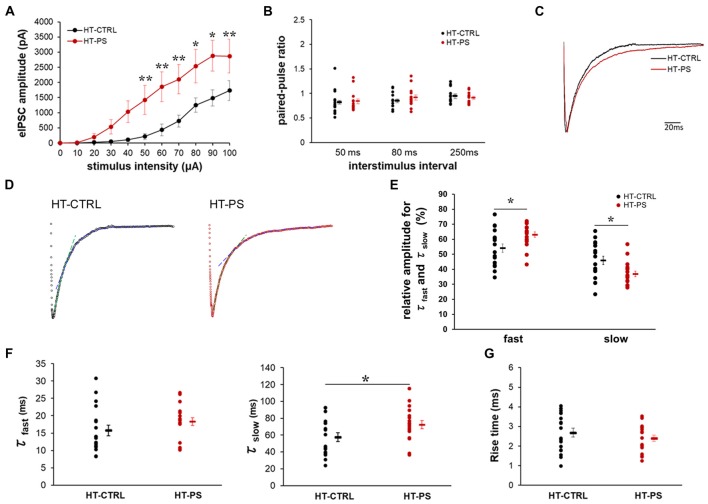
Altered excitability of the inhibitory network and slower decay kinetics of evoked inhibitory post-synaptic current (IPSC) in glutamate decarboxylase (GAD)67^+/GFP^ mice with PS. **(A)** Relationship of increasing stimulus intensities and evoked inhibitory post-synaptic currents (eIPSCs) amplitudes. Current response amplitudes in GAD67^+/GFP^ mice with PS were significantly increased at intensities of 50–100 μA compared with controls (*P < 0.05, ** P < 0.01 Mann-Whitney *U*-test at each intensity point; *n* = 18 cells from seven HT-CTRL mice and *n* = 18 cells from seven HT-PS mice). **(B)** Summary graph of (paired-pulse ratio (PPR); eIPSC2/eIPSC1) measurements obtained from paired stimulus with 50, 80 and 250 ms intervals, respectively (*t*-test). **(C)** Representative trace of normalized IPSCs evoked by focal stimulation recorded from a layer V pyramidal neuron of medial prefrontal cortex (mPFC) of HT-CTRL (black) and HT-PS (red). **(D)** Representative examples of double exponential fitting of evoked inhibitory post-synaptic currents (eIPSCs) decay. Dashed lines indicate the fast (green) and slow (blue) exponential fitting of the representative trace of eIPSC. **(E)** Plot shows the percentage of relative amplitudes corresponding to the fast and slow decay phases between the two groups. *P < 0.05, *t*-test.** (F)** Plot shows the decay time constant (τ_fast_ and τ_slow_) of eIPSC. *P < 0.05, *t*-test. **(G)** Summary graph for rise time (10%–90%) of eIPSC. No change was detected in the 10%–90% rise time between the two groups.

To ascertain the alterations in GABAergic signaling as a consequence of changes in specialized ECM components associated with GABAergic synapses in our model, we examined the eIPSC characteristics in GAD67^+/GFP^ mice with or without PS. We evaluated characteristics of the inhibitory signaling on layer V pyramidal neurons of mPFC. The relationship between the eIPSC amplitude and stimulus intensity is shown in Figure 5A. HT-PS neurons responded to low stimulus intensities of 10–30 μA, which was absent in HT-CTRL neurons. Thus, threshold stimulus intensity for eIPSC events was significantly reduced in HT-PS. In addition, we found that eIPSC amplitudes were significantly greater in the HT-PS group at stimulus intensities ranging from 50 μA to 100 μA compared with HT-CTRL. This enhancement of eIPSC amplitude may be because of changes in release changes in release probability (P*r*) of GABA from the presynaptic terminals. To assess this possibility, we performed a paired-pulse protocol at a stimulus intensity evoking 60%–70% of maximal response at 50 ms, 80 ms and 250 ms ISI for each neuron. However, the analysis of PPR for HT-CTRL and HT-PS showed no significant difference at 50 ms, 80 ms and 250 ms ISI (Figure 5B). Next, we examined the decay kinetics and 10%–90% rise time to determine whether there were significant changes between the groups. The representative normalized eIPSC traces for HT-CTRL and HT-PS are shown in Figure 5C. A detailed evaluation of decay kinetics of evoked responses was performed by double exponential fitting. Representative examples of fitting of eIPSC decay are shown in Figure 5D. The correlation coefficients (R-square) were calculated to be 0.76 for HT-CTRL and 0.81 for HT-PS respectively, suggesting goodness of fit when using double exponential model. A comparison of the obtained decay rates (τ_fast_ and τ_slow_) indicated a significantly prolonged τ_slow_ in the HT-PS group as observed in Figure 5F (in ms; τ_fast_ HT-CTRL: 15.8 ± 1.5; HT-PS: 18.3 ± 1.1; *P* = 0.057 by *t*-test; τ_slow_: HT-CTRL: 57.5 ± 5.2; HT-PS: 72.3 ± 4.8; *P* < 0.05 by *t*-test). In addition, comparison of the percentage of relative amplitudes between the two groups corresponding to both the fast and slow decay phases were observed to be significantly different as shown in Figure 5E (in %; rel. amp._fast_ HT-CTRL: 54.11 ± 2.82; HT-PS: 63.11 ± 1.81; rel. amp._slow_: HT-CTRL: 45.89 ± 2.82; HT-PS: 36.89 ± 1.81; *P* < 0.05 by *t*-test). However, quantitative analysis of the 10%–90% rise time indicated no significant difference between groups (Figure 5G). Also, to rule out the effect of genotype alone on inhibitory signaling, we evaluated eIPSC characteristics in WT-CTRL. A comparison of eIPSC characteristics between WT-CTRL and HT-CTRL revealed no significant differences (Supplementary Figure S2). These results further support the prerequisite of this specific gene–environment interaction for consequentially inducing alterations of specialized ECM structures associated with GABAergic synapses thereby affecting the inhibitory signaling to layer V pyramidal neurons of the mPFC.

### Change in Basic Properties of Spontaneous Inhibitory Signaling in GAD67^+/GFP^ Mice With PS

**Figure 6:**
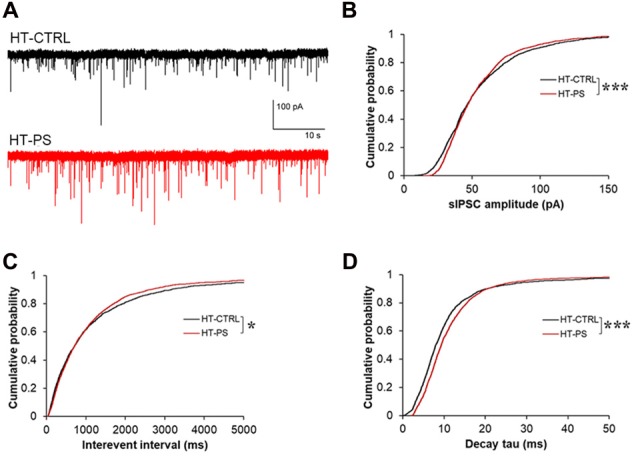
Change in basic properties of spontaneous inhibitory signaling in GAD67^+/GFP^ mice with PS. **(A)** Representative traces showing spontaneous IPSC (sIPSC) recorded in layer V pyramidal neurons at –70 mV from mPFC of HT-CTRL (black) and HT-PS (red) mice. **(B–D)** Cumulative plots of sIPSC amplitude **(B)**, interevent interval **(C)** and decay tau **(D)**. *P < 0.05, ***P < 0.001 Kolmogorov-Smirnov test; *n* = 17 cells from five HT-CTRL mice and *n* = 17 cells from five HT-PS mice.

Given the lower stimulus threshold intensity and increased amplitude of eIPSC, we hypothesized that the excitability of GABAergic interneurons might be increased, which would be reflected in sIPSCs in mPFC pyramidal neurons. In order to measure basic properties of sIPSC, whole-cell recordings of layer V pyramidal neurons were performed. Representative sIPSC traces for HT-CTRL and HT-PS are shown in Figure 6A. We observed significant difference in distribution of sIPSC amplitude (*P* < 0.001; Figure 6B), in which lower and higher amplitude fraction apparently increased and decreased in stressed mice. Also the reductions in interevent interval of sIPSC events were observed (*P* < 0.05; Figure 6C). Next, we measured the decay tau in the sIPSCs and observed that the time constant was significantly longer in comparison with control animals (*P* < 0.001; Figure 6D). The alterations of the sIPSC characteristics in addition to the eIPSC changes reported in the HT-PS group suggest inhibitory synapses on layer V pyramidal neurons of the mPFC undergo remodeling paralleling the alteration of ECM and loss of PV neurons triggered by the gene–environment interaction.

## Discussion

In the present study, we used a gene–environment interaction mouse model with *Gad1* abnormalities as a genetic risk and PS as an environmental risk to investigate whether abnormalities in the ECM would alter the function of GABAergic synaptic transmission relevant to the pathogenesis of neuropsychiatric diseases. Our findings show alterations of GABA neuron-associated ECM in the mPFC, i.e., decreased PNNs densities along with the loss of PV neurons and decrease in the glycosylation of α-DG in GAD67^+/GFP^ offsprings subjected to PS. Functional evaluation of GABAergic inhibitory input to layer V pyramidal cells in the mPFC of GAD67^+/GFP^ mice with PS indicated the threshold stimulus intensity for eIPSC was reduced, the amplitude was increased without changes in the PPR, and the decay rates of eIPSCs were prolonged. The changes in eIPSC were generally concomitant with a marked increase in sIPSC frequency, amplitude and decay time constant.

Altered expression of various GABA-related proteins and/or decreased GABA concentrations in multiple regions of the brain have been reported in postmortem studies of human patients with psychiatric disorders (Akbarian et al., 1995; Yip et al., 2007; Curley et al., 2011; Berretta et al., 2015; Dong et al., 2015; Frankle et al., 2015; Chung et al., 2016; Enwright et al., 2016). In particular, altered levels of *GAD1* transcripts encoding GAD67, were reported in the prefrontal cortex of schizophrenic subjects (Volk et al., 2000). Dysfunctions of PV interneurons were identified in both postmortem human clinical studies and rodent models suggesting a major pathophysiological mechanism constituting these changes as an underlying etiology of psychiatric disorders (Beasley et al., 2002; Lewis et al., 2012; Marín, 2012; Enwright et al., 2016). In addition, immature developmental gene expression profiles of PV interneurons have been reported in ASD and schizophrenic patients (Gandal et al., 2012). These results suggest disrupted inhibitory control in psychiatric disorders. In our previous study, we showed that decrements of PV-positive GABAergic interneurons in the mPFC of GAD67^+/GFP^ offspring exposed to PS (Uchida et al., 2014). Moreover, GAD67 decrements induced by genetic manipulation in our model mimic the classical features frequently observed in multiple brain regions of patients with psychiatric disorders. Therefore, our animal model might be useful to investigate the underlying mechanisms involved in the pathogenesis of such psychiatric disorders.

The PV neurons are enwrapped by PNNs as they mature (Härtig et al., 1992). Recently, it was reported that the maturation of FS properties of perisomatic inhibitory neurons in the mPFC occurs around postnatal day (P) 12 ± 2 (Miyamae et al., 2017). This FS property of PV interneurons parallels with their being enwrapped by PNN. Thus, development of the inhibitory network in combination with ECM changes is essential for the function of mPFC. In the present study recapitulating a neurodevelopmental approach to the pathogenesis of psychiatric diseases, we observed that the numbers PV+ neurons associated with WFA+ and ACAN+ PNNs were reduced in the mPFC of HT-PS (Figures 1, 2). This reduction of PV neurons might be caused by the reduced neurogenesis of GABAergic interneurons in MGE as previously reported (Uchida et al., 2014). Decrease in PNN could in turn enhance the susceptibility of presenting PV+ neurons in the mPFC to oxidative damage because of their high metabolic requirements for FS function (Cabungcal et al., 2013). Since neither WFA nor ACAN intensity was reduced in remaining PV+ neurons and any other cell type (see Figures 1, 2), the decrease in PNNs might be the result of loss of PV+ neurons enwrapped by PNNs, but not of PNNs itself. Several lines of evidences suggested that the maturation of PNNs surrounding the PV neurons coincides with the closure of the critical period, a window of heightened plasticity during brain development, and that the function of PNNs in neuronal plasticity involves regulating synapse stabilization and limiting synaptic differentiation and formation (Pizzorusso et al., 2002; Carulli et al., 2016; Favuzzi et al., 2017). Therefore, the decreases in the total tissue PNNs might cause substantial recapture of plasticity on the function of inhibitory networks.

Another component of ECM associated with inhibitory synapses is dystroglycan (DG). Despite its importance in various diseases with muscular dystrophies, the role of the DG complex in the nervous system has only recently been highlighted. To date, the presence of α-DG has been confirmed in the postsynaptic terminals of GABAergic synapses and was reported to modulate the plasticity of synaptic function (Knuesel et al., 1999; Lévi et al., 2002). In the mPFC of our model, we found the glycosylation of α-DG was significantly decreased. Furthermore, disruption of the mRNA expression level of *Fktn*, one of the putative glycosyltransferases, was observed in the mPFC (Figure 4C). The reduction of the glycosylation of α-DG suggests the possibility of changes in the ligand-binding and structure of the extrasynaptic space around GABAergic synapses. Taking the interneuron-specific role of α-DG into consideration will help elucidate the mechanisms underlying the dysfunction of GABAergic inhibition in our model. A recent study (Früh et al., 2016) demonstrated that the neuronal α-DG plays an essential role in the trans-synaptic signaling necessary for the formation and maintenance of functional axon terminals by cholecystokinin (CCK)-positive GABAergic basket cells, which specifically target the perisomatic region of principal neurons. The α-DG conditional knock-out rendered the complete loss of CCK-positive terminals forming synapses on pyramidal neurons. We speculate that the reduction in FUKUTIN-mediated glycosylation of α-DG may affect the binding of extracellular ligands like laminin, neurexin amongst others, and hence its transsynaptic linker role. This potential alteration of the transsynaptic linker role of the α-DG might facilitate the apposition of the inhibitory presynapse and postsynaptic clustering of GABA_A_R, and thus might facilitate GABAergic inputs to the layer V pyramidal neurons. Therefore, the reduction of glycosylation pattern of α-DG in HT-PS may reverse the maintenance leading to remodeling of CCK+ terminals. Also, it could be a compensatory effect for the loss of PV neurons and their associated PNNs, because observed changes in the evoked IPSCs and sIPSCs were compatible with such compensation. This may consequently affect GABAergic functions in the mPFC.

In this study, the input/output characteristics indicated a reduced eIPSC threshold and increased amplitudes in the mPFC of HT-PS without affecting PPR (Figure 5), suggesting such changes in the ECM structures associated with inhibitory synapses may affect the overall excitability of inhibitory network in the mPFC of HT-PS mice. This was further evidenced in the increase in frequency of sIPSC events (Figure 6) and may suggest compensatory increase of presynaptic inputs. As the electrically evoked IPSCs reflect activation of different types of interneurons with terminals in both the dendritic and perisomatic compartments of the layer V pyramidal neurons, it would be interesting to further investigate the differential effect of interactions and *Gad1* heterozygosity on excitability of different types of interneurons. A multitude of factors may potentially contribute to this altered excitability of the inhibitory network. For instance, changes in ion channel conductance as well as density may contribute to reduction of the passive conductance of the membrane; change the action potential (AP) threshold, AP propagation along the axon, thereby increasing the excitability of interneurons. Another possibility would be the altered charge dissipation or reduced membrane screening due to the ECM loss during an electrical stimulation, resulting in enhanced excitability of the inhibitory network in the mPFC of HT-PS. Yet another factor affecting the excitability of the inhibitory network may be changes in axonal myelination of PV interneurons. Previous report suggested that majority of GABAergic axon terminals in the cortex are positively labeled for PV. The myelin sheath’s role in energy efficient propagation of depolarization along the axon is critical to the high frequency firing exhibited by this subtype of GABAergic interneurons (Micheva et al., 2016). As our model reports a reduction of PV interneuron neurogenesis, as a compensatory mechanism changes in myelination of the remaining PV interneurons could enhance their excitability.

As the amplitude of both eIPSC and sIPSC in HT-PS group were increased significantly, postsynaptic changes in the GABA_A_ receptor subunit and their clustering are also likely. These changes may reflect a compensatory mechanism arising due to the reduced GAD67 levels in the already decreased population of PV interneurons enwrapped by PNNs. Indeed, postsynaptic GABA_A_ receptor changes resulting from reduced GABA levels have been observed in a previous report (Lazarus et al., 2015). Alternatively, the diffusion of neuroactive substances (ions, neurotransmitters) in the extracellular space dependent on the structure and properties of the ECM is another issue (Syková, 2004; Vargová and Syková, 2014). Because these ECM structures have a high molecular weight, they might occupy the synaptic cleft volume and thereby regulate the diffusion of GABA. This in turn may regulate dwell time (Perrais and Ropert, 2000) and contribute to the increased amplitude of eIPSC and sIPSC. As previously reported, the function of GABA uptake becomes the rate limiting step in governing the kinetics of eIPSC in granule cells of the dentate gyrus (Draguhn and Heinemann, 1996). Since significant prolongation of τ_slow_ was observed, GABA uptake could be decreased in HT-PS. This observation is further supplemented with prolonged decay tau values estimated for sIPSC events. The increased likelihood of two distinct mechanisms contributing to the slower decay kinetics in HT-PS group requires identifying their specific components, which may be very challenging at present. However, the significantly altered relative amplitude fractions of eIPSC corresponding to fast and slow phases of decay suggest that a combination of altered GABA_A_ receptor subunit prolonging fast decay (Gingrich et al., 1995) and slower GABA uptake prolonging slow decay by GABA transporters (GAT) could underlie observed phenomenon.

**Figure 7:**
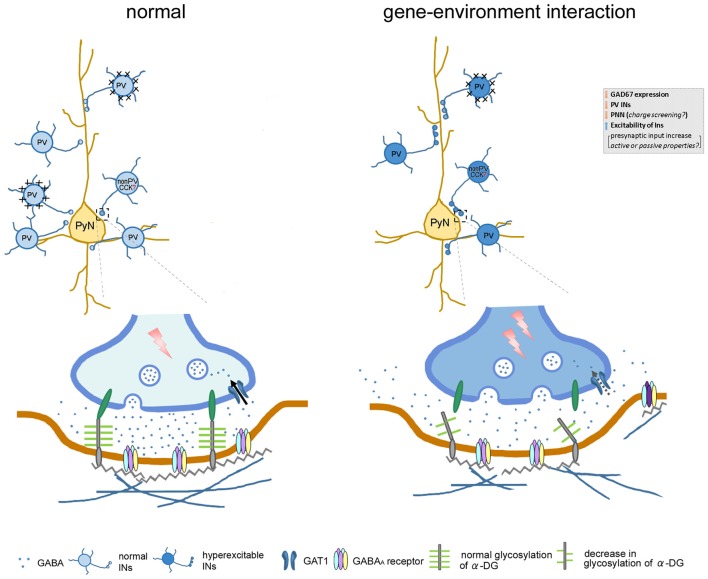
Cartoon illustrates the affect gene–environment interaction on neuronal morphology, along with alteration in function and pathophysiology of GABAergic signaling within neuronal network. In our gene–environment interaction animal model, *GAD67* expression and GABA synthesis was reduced in interneurons. The decreased density of PV neurons and their enwrapping PNN might be compensated by enhanced GABAergic functions. Decrement of glycosylation of α-DG impairs the link between pre- and postsynaptic sites by connecting with the cell adhesion molecules embedded in the pre- and postsynaptic membranes. Alteration in these GABAergic neuron-associated extracellular matrix consequently may: (i) increase presynaptic input of remaining PV and non-PV (putative cholecystokinin) interneurons; (ii) affect diffusion of GABA out of the synaptic cleft to the extrasynaptic region; (iii) regulate clustering of GABA_A_ receptors or subunit composition; and (iv) affect function of GABA transporter (GAT) responsible for the re-uptake of GABA at synapses. These changes may in turn increase spontaneous firing of inhibitory network, decrease the threshold of stimulus of evoked IPSC, regulate decay time constant and contribute to the increased amplitude of evoked inhibitory post-synaptic currents (eIPSCs). Abbreviations: CCK, cholecystokinin; INs, interneurons; PyN, pyramidal neuron; PV, parvalbumin.

In conclusion, *Gad1* abnormalities may be a genetic risk factor that could interact with environmental risk factors such as PS to induce loss of PV neurons and alteration of ECM (PNNs and α-DG). These alterations may underlie the observed changes in synaptic inhibition in the layer V of mPFC (Figure 7), thereby affecting the functional output of the mPFC, essential for higher cognitive functions. Since both *Gad1* abnormalities and PS are risk factors and decrements in PV and ECM in the mPFC are phenotypes of psychiatric disorders, further studies to delineate in depth molecular and physiological mechanisms and behavioral phenotypes, as well as their onset time point in our model remain as future challenge.

## Author Contributions

TW and AS performed experiments. YY generated mice. AF designed the project. TW and AF wrote the manuscript. TA contributed to the revision process, especially about electrophysiological analyses and revising the manuscript.

## Conflict of Interest Statement

The authors declare that the research was conducted in the absence of any commercial or financial relationships that could be construed as a potential conflict of interest.

## Footnotes

^1^https://www.ncbi.nlm.nih.gov/tools/primer-blast/

^2^https://www.ncbi.nlm.nih.gov/BLAST/
