# Supplementary material for: Alterations of GABAergic Neuron-Associated Extracellular Matrix and Synaptic Responses in Gad1-Heterozygous Mice Subjected to Prenatal Stress
Source: Front Cell Neurosci. 2018 Sep 5;12:284. doi: 10.3389/fncel.2018.00284 (PMC6133952; doi:10.3389/fncel.2018.00284)
Supplement: Supplementary file 1 [file Data_Sheet_1.docx]

**Supplementary Material**

**Alterations of GABAergic neuron-associated extracellular matrix and synaptic responses in *Gad1*-heterozygous mice subjected to prenatal stress**

Tianying Wang^1†^, Adya Saran Sinha^1†^, Tenpei Akita^1^, Yuchio Yanagawa^2^, Atsuo Fukuda^1,3^**^*^**

*****Correspondence: Atsuo Fukuda: [axfukuda@hama-med.ac.jp](mailto:axfukuda@hama-med.ac.jp)

**Supplementary Figures**


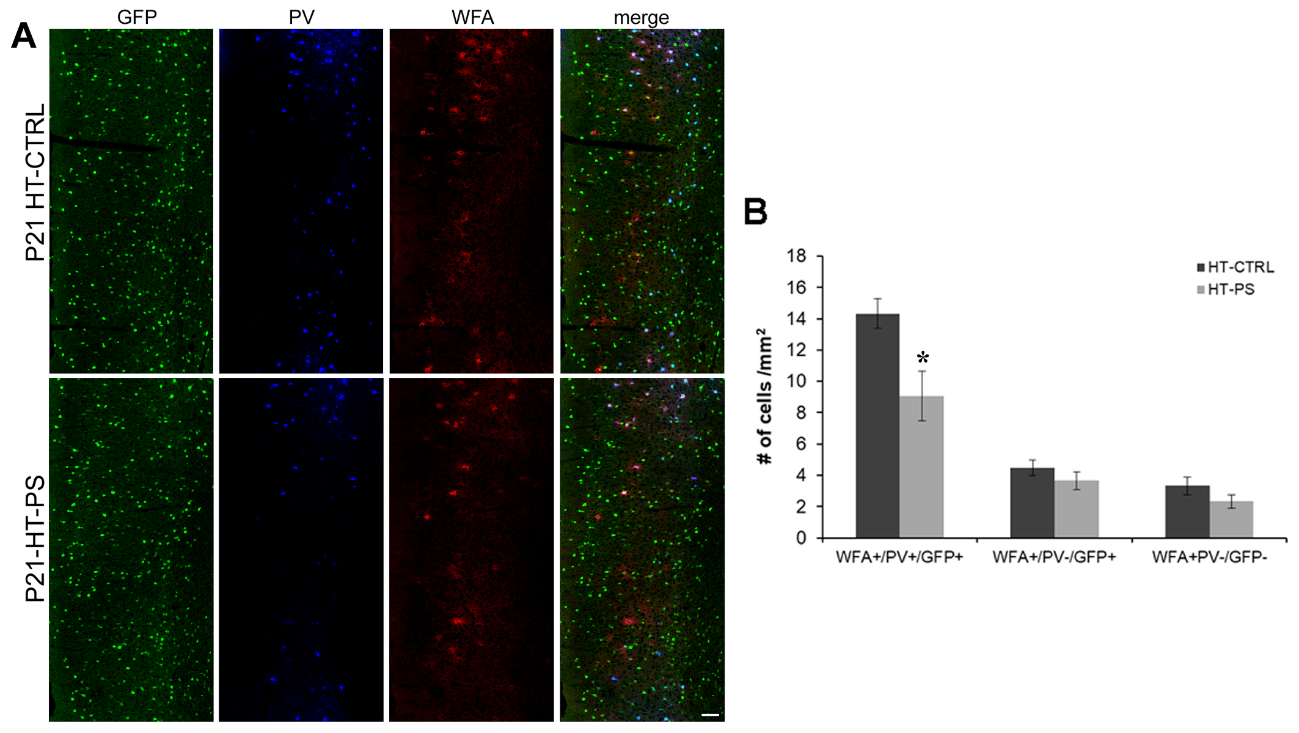


**Supplementary Figure 1. Unchanged densities of PV-negative cells enwrapped by PNNs in the medial prefrontal cortex (mPFC) of prenatally stressed GAD67^+/GFP^ mice at P21.** (**A**) Representative immunostaining images of GFP (green), PV (blue) and WFA (red) from coronal sections of the P21 GAD67^+/GFP^ control and stressed brain. (**B**) Quantitative analysis of cell densities. The number of PV+/GFP+/WFA+ cells were significantly decreased in mPFC in subjects with PS (n=7 slices from 3 mice) compared with that in control (n=8 slices from 4 mice; *t*-test, **P*<0.05). There was no significant difference in the cell density of PV-negative GABAergic neurons (WFA+/PV−/GFP+) and non-GABAergic cells (WFA+/PV−/GFP−) enwrapped by PNN between control and PS mice.

#
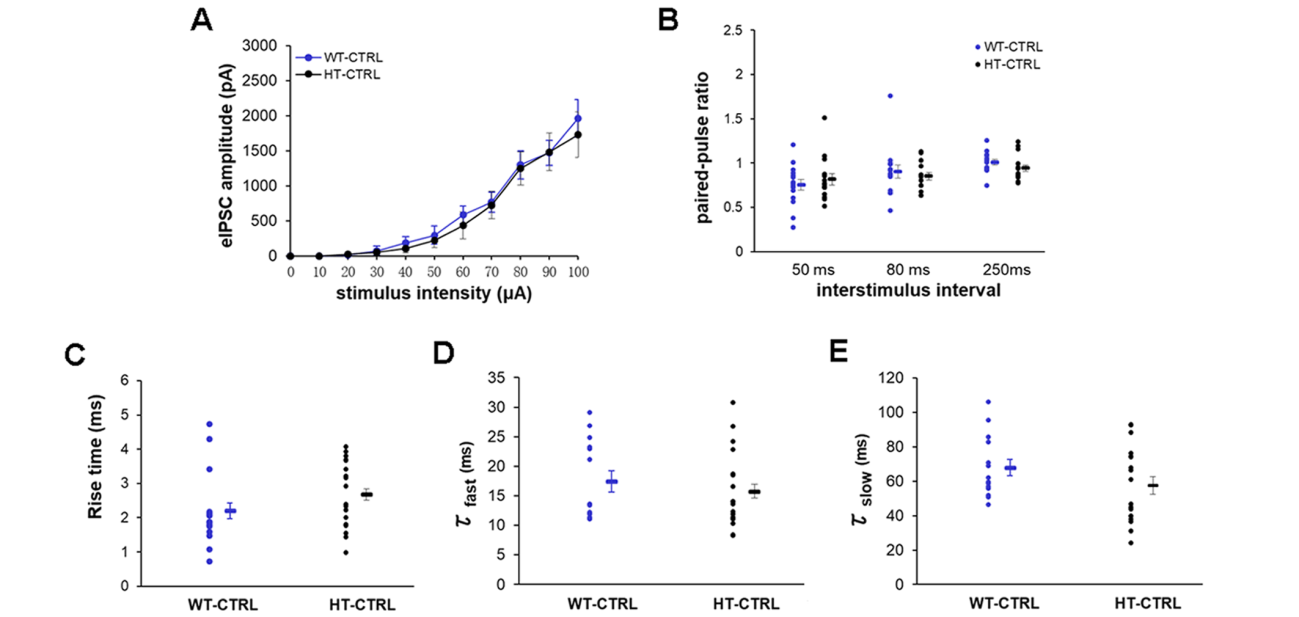
Supplementary Figure 2. Identical inhibitory network and kinetics of eIPSC between WT-CTRL and HT-CTRL mice. (A) Relationship of increasing stimulus intensities and eIPSC amplitudes. Current response amplitudes in HT-CTRL mice were similar compared with WT-CTRL (Mann-Whitney *U*-test at each intensity point; n = 14 cells from 4 mice of WT-CTRL; n = 18 cells from 7 mice of HT-CTRL). (B) Summary graph of paired-pulse ratio (eIPSC2/eIPSC1) measurements obtained from paired stimulus with 50, 80 and 250 ms intervals, respectively. There was no significant change in PPR of eIPSC (*t*-test). Summary graph for rise time (10%–90%) (C), τ_fast_ (D) and τ_slow_ (E) of eIPSC. No change was detected in the eIPSC kinetics between the two groups.
